# A holistic view of facilitators and barriers of electronic health records usage from different perspectives: A qualitative content analysis approach

**DOI:** 10.1177/18333583231178611

**Published:** 2023-07-07

**Authors:** Anna Griesser, Sonja Bidmon

**Affiliations:** Alpen-Adria-Universitaet Klagenfurt, Austria

**Keywords:** electronic health records, patient perspective, physician perspective, barriers, facilitators, holistic view, health digitalisation, Austria, qualitative content analysis, health information management

## Abstract

**Background:**

Electronic health records (EHR) are seen as a promising endeavour, in spite of policies, designs, user rights and types of health data varying across countries. In many European countries, including Austria, EHR usage has fallen short when compared to the deployment plans.

**Objective:**

By adopting a qualitative approach, this research aimed to explore facilitators and barriers experienced by patients and physicians across the entire EHR usage process in Austria.

**Method:**

Two studies were conducted: In Study 1, discussions were held with four homogeneously composed groups of patients (*N* = 30). In Study 2, eight expert semi-structured interviews were conducted with physicians to gain insights into potential facilitators and barriers Austrian physicians face when utilising personal EHR.

**Results:**

A wide range of barriers and facilitators were identified along the entire EHR usage spectrum, emerging on three different levels: the micro-level (individual level), the meso-level (level of the EHR system) and the macro-level (level of the health system). EHR literacy was identified as a booster to support EHR adherence. Health providers were identified as crucial gatekeepers with regard to EHR usage.

**Conclusion:**

The implications for mutual benefits arising out of EHR usage among the triad of health policymakers, providers and patients for both theory and practice are discussed.

## Introduction

The process of digitalisation in health has led to new approaches aimed at patient care and networking with and between health professionals (Meister et al., 2018), accelerated by, for example, challenges with regard to long-time care or changed demographics (Evans, 2016; Kreps and Neuhauser, 2010). Patient-related electronic health records (EHR) are viewed as a promising instrument, ranging from ‘stand-alone’ to connected and integrated approaches (Tenforde et al., 2011). These health data rely on web-based applications, which can be shared among the triad of health policymakers, health providers and patients (Essén et al., 2018; Tavares and Oliveira, 2016), and can support a transparent exchange of health data and improve quality of care (Menachemi and Collum, 2011). The term ‘EHR’ should be differentiated from electronic medical records (EMR), which contain the standard medical and clinical data gathered in one specific healthcare institution. EHR extend beyond and include a more comprehensive patient history than EMR (McMullen et al., 2014).

To date, European stakeholders have been motivated to advance the development of EHR systems for different reasons (Damschroder et al., 2007), including facilitation of cross-border interoperability in the European Union, pushed by a desire to improve medical and nursing quality, promote medical research into health challenges and strive towards sustainability and efficiency of national health system (NHS; George et al., 2013). For example, frontrunners Denmark and Estonia, with approximately two million visits by patients per month, are well advanced in terms of EHR implementation (Rahbek Norgaard, 2013), while countries such as France (Burnel, 2018), the United Kingdom (Bonomi, 2016) and Austria (Hoerbst et al., 2010) have experienced difficulties that have halted their plans. The French EHR programme was relaunched in 2016, after the initial adoption of the nationwide framework had been historically poor (Séroussi and Bouaud, 2018). In 2010, the NHS in the United Kingdom faced numerous difficulties linked to the rollout plan, including software problems and absence of deadlines (Bonomi, 2016). EHR have great potential but interoperability has only been successful within and across various national health sectors, while not yet possible in others (European Commission, 2021).

There has also been much debate about the choice to ‘opt-out’ or to explicitly ‘opt-in’ to maintain sharing of confidential information on the national database (Powell et al., 2006). ‘Opt-in’ is the process used when positive action is required to subscribe a patient to an EHR system. ‘Opt-out’, on the other hand, means that each patient receives a login to the EHR system, and individuals have to take action on their own if they want to withdraw from the EHR system. Both systems differ with regard to the level of informed consent. An ‘opt-in’ system makes it more likely that individuals have given their explicit consent to create an EHR (Pearce and Bainbridge, 2014 cited by Torrens and Walker, 2017). The default setting of an ‘opt-out’ system is characterised by presumed consent, which can expose individuals to greater risks of harm related to EHR use. In Australia, a country that switched from an ‘opt-in’ system to the present ‘opt-out’, patients have additional strengthened privacy protections guaranteed (Torrens and Walker, 2017). Austria is another example of an ‘opt-out’ system, as EHR is automatically created for each Austrian inhabitant. However, the principals (Federal, state and local governments), who established the EHR system ‘ELGA’ (the German term for ‘EHR’) in 2009, have faced resistance from patients, with fewer than 10,000 EHR system accesses counted per month (ELGA GmbH, 2021).

Historically, physicians have been the specific gatekeepers in their role of facilitating access for patients to specialised care (Brooks et al., 2013). Lauckner and Whitten (2016) demonstrated that physicians were the most essential gatekeepers for telemedicine. Physician engagement has also been identified as both the enabler to increase innovation adoption and the greatest potential barrier to be overcome to expand innovation services. In addition, a broad literature base exists in other scientific domains (e.g. marketing), which have studied innovation adoption from the patients’ perspective (i.e. Smith et al., 2020).

With regard to the range of theoretical foundations applied in EHR studies, the most widespread are ‘technology acceptance’ approaches (e.g. Cherif et al., 2021). Most popular is the original ‘Technology Acceptance Model’ (TAM) developed by Davis (1985), which conceptualises perceived ease of use and perceived usefulness (PU) as central antecedents of technology acceptance (Davis, 2005). Since then, several extensions of the original TAM have been developed, such as the Unified Theory of Acceptance and Use of Technology (UTAUT) (Venkatesh et al., 2003). The more sophisticated version of the TAM, UTAUT2 (Venkatesh et al., 2012) has led to a proposal for a multilevel framework of technology acceptance and use (Venkatesh et al., 2016). However, Tamilmani et al. (2021) conducted a systematic literature review on 650 UTAUT2-based studies and concluded that adding new constructs and associations with higher-order moderation effects led to low parsimony of the UTAUT2, which is a major drawback when compared to the original TAM.

The aim of the current study was to investigate possible barriers and facilitators of EHR usage, exemplified by the Austrian EHR system for the two major user groups: patients and physicians (Halmdienst et al., 2022).

## Theoretical background and research questions

A considerable body of literature on EHR usage has been published, from both patients’ and physicians’ perspectives (e.g. Ancker et al., 2015; Powell, 2017), with a broad range of facilitators highlighted, such as digital literacy (Alanazi et al., 2020), and education and training as a primary facilitator (Mold et al., 2015). Furthermore, a high level of usability of the system (Zarcadoolas et al., 2013) and compliance with security requirements, integration and sharing of EHR (Rezaeibagha et al., 2015) have been shown to foster usage for patients as well as for physicians. In contrast, several barriers for patients have also been found, including a lack of PU for the EHR system (Cowell, 2002), or a negative attitude or simply no interest towards new technologies (Honeyman et al., 2005). In particular, two studies among physicians conducted in 2016 and 2021 revealed that the majority of physicians surveyed had already experienced a fear of EHR-related administrative burden, increased workload, as well as feeling uncomfortable with regard to a possible surveillance without benefiting from using EHR in their day-to-day practice (Jamoom et al., 2016; Rahal et al., 2021).

However, too little attention has been paid to taking a holistic view of the entire EHR usage process, which initially begins with awareness raising, then proceeds to adoption, followed by usage, and the evaluation of the final consequences of EHR usage (Larsen, 2003). The core element of a holistic approach is to understand all aspects of patients’ and physicians’ needs: psychological, physical as well as social (Crameri et al., 2022). In terms of EHR, a holistic understanding could help to encourage EHR usage on a broader scale (Smith et al., 2020; Strandberg et al., 2007). For this study, the ‘adoption’ step has been replaced with ‘willingness to use’, as we believe this term is a better match for this stage and more meaningful within the context of EHR usage.

As the first step in our research, we undertook a systematic literature review from the patients’ perspective (Griesser and Bidmon, 2023). The current approach takes this a step further and aims to develop in-depth knowledge on barriers and facilitators of both patients’ and physicians’ EHR usage. Furthermore, it is imperative to focus on the physicians’ perspective to emphasise the relevance of a possible gatekeepers’ role in the acceptance of EHR usage. In this study, the following general research questions were explored: (1) what facilitators do patients and physicians perceive regarding EHR in each step of EHR usage? and (2) what barriers do patients and physicians perceive regarding EHR in each step of EHR usage?

## Method

### Study 1

In this study, a qualitative exploratory design using focus group discussions was chosen. Four groups, homogeneous with regard to user experience and age, were drawn from *N* = 30 participants: experienced users included seven participants ⩽45 years old and nine aged >45 years; and non-users included seven participants ⩽45 years old and seven aged >45 years. Characteristics of the patient participants are shown in Table 1. A semi-structured, pilot-tested interview guideline was used. Focus groups were conducted in September and October 2021, producing total 262 minutes of comments, which were transcribed verbatim and analysed (average: 60 minutes). For those groups without user experience, a brief introduction to the Austrian version of the EHR platform was given.

### Study 2

To investigate the barriers and facilitators faced by physicians in Austria, another qualitative research setting was applied. Eight physicians were recruited for this study, with participant characteristics shown in Table 2. Primary data were gathered through eight semi-structured interviews, producing a total of 216 minutes. The interviews were performed from February to March 2022 and were held in German (i.e. the native language of the target group of the respondents).

#### Design of the study

Study 1 aimed to analyse the patient perspective using focus group discussions, while Study 2 examined the physician’s perspective. All participants were recruited using purposive sampling (see, e.g. Ninaus et al., 2015). By taking a segmentation perspective, we differentiated between patients’ EHR usage experience, gender, age and educational background and physicians’ gender, age, medical specialty and setting. All collected data during the research process underwent a translation process to ensure rigour in translation. The method included transcribing verbatim in the original language in which data were collected, using an English native speaker who was linguistically competent and culturally sensitive in both languages and moving back and forth between raw and translated data to guarantee consistency (Mohamad et al., 2020).

#### Data analysis

Following the procedure of qualitative content analysis as proposed by Mayring and Fenzl (2014), the first author read the transcripts several times. During the analysis, the main topics were identified deductively (approaching the data with some preconceived themes based on existing literature), as well as inductively (allowing the data to determine the categories), with subsumption of categories or formulating new categories. After several feedback loops, a revision of categories was prepared, with a formative check of reliability and a final coding system. After summarising and labelling key issues as codes across all transcripts, the codes were sorted into main and subcategories. The software ‘MAXQDA’ was used as a supporting tool for data analysis. In addition, one transcript of the focus groups was reviewed independently by the first author and one bachelor’s degree student looked for discrepancies and the level of agreement. Discrepancies were solved by discussion between the two authors and the bachelor’s degree student.

Thus, the current approach investigated in-depth knowledge on barriers and facilitators of patients’ and physicians’ EHR usage along the entire usage process: from the first moment of awareness, to willingness to use and the existing user experience. In addition, physicians could be identified as EHR gatekeepers that could set the process in motion:

Step 0: Facilitators and barriers related to physicians as EHR gatekeepers
Step 1: Facilitators and barriers of awareness of EHR
Step 2: Facilitators and barriers of willingness to use EHR
Step 3: Facilitators and barriers of usage experience with EHR

#### Ethics approval

Group discussions and expert interviews received prior approval from the authors’ university ethics committee (Institutional Review Board for Research Ethics at the University of Klagenfurt (ER-AAU); ID: 2022-001 & ID: 2021-039). Informed written consent was obtained from all individual participants included in the study.

## Results

### Sample description (Studies 1 and 2)

**Figure 1.**
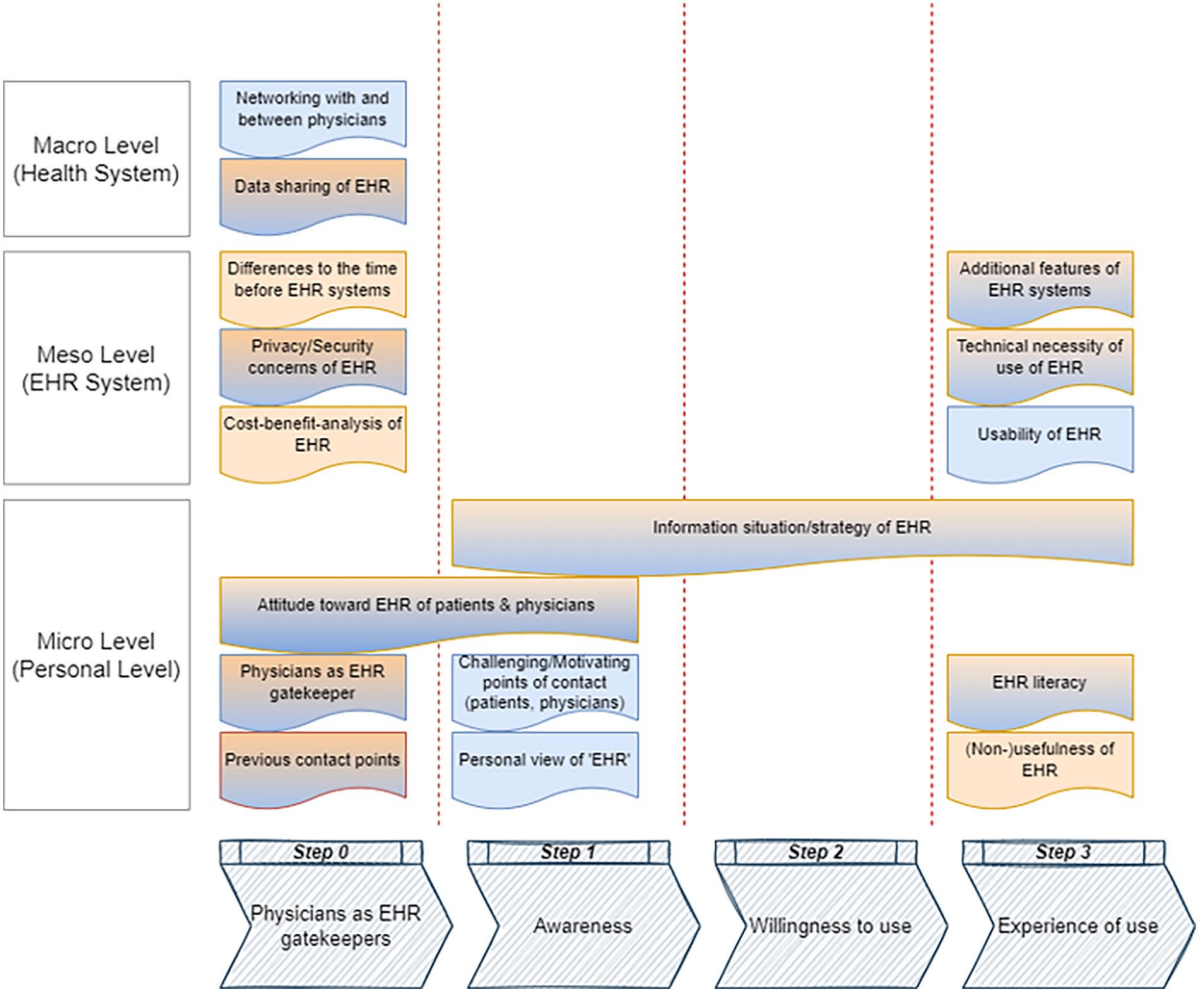
Code system of deductively and inductively derived categories of facilitators and barriers (blue: patient perspective; orange: physician perspective; and blue and orange: consistent view).

For Study 1, we decided to differentiate between the two target groups of patients with or without EHR usage experience. Table 1 reveals the sample description separated according to the subgroups of EHR users. For Study 2, eight in-depth interviews with physicians were conducted. Participant details are provided in Table 2. Barriers and facilitators were identified on the macro-level of the healthcare system, the meso-level of the EHR system, and the micro-level on the personal level. In all, 16 barriers and facilitators were synthesised (see Figure 1). Findings in Figure 1 from the patient’s perspective are marked in blue and the physician’s perspective are in orange. In the case of a consistent view of patients’ and physicians’ perspectives, the coloration is orange and blue.

**Table 1.** Description of patients participating in the focus groups according to subgroups: Discussion groups of EHR users (Groups 1 and 2) versus non-users (Groups 3 and 4).

Group 1: EHR users aged up to 45 years					Group 2: EHR users aged 46 years and above				
ID	Gender	Age	Highest level of education	EHR usage experience in years	ID	Gender	Age	Highest level of education	EHR usage experience in years
U1	Female	29	University	2 (2019)	U8	Male	52	High school	6 (2015)
U2	Male	30	University	< 1 (2021)	U9	Male	47	High school	1 (2020)
U3	Female	33	University	3 (2018)	U10	Male	70	High school	7 (2014)
U4	Female	25	University	3 (2018)	U11	Male	53	University	2 (2019)
U5	Female	26	University	< 1 (2021)	U12	Male	61	University	6 (2015)
U6	Female	29	High school	6 (2015)	U13	Male	55	University	< 1 (2021)
U7	Male	26	High school	< 1 (2021)	U14	Female	46	University	2 (2019)
					U15	Female	57	High school	7 (2014)
					U16	Male	47	University	6 (2015)
Group 3: EHR non-users aged up to 45 years					Group 4: EHR non-users aged 46 years and above				
ID	Gender	Age	Highest level of education	EHR usage experience in years	ID	Gender	Age	Highest level of education	EHR usage experience in years
N1	Male	35	University	None	N8	Male	55	University	None
N2	Female	29	University	None	N9	Male	54	University	None
N3	Female	23	University	None	N10	Male	68	University	None
N4	Female	27	University	None	N11	Female	50	High school	None
N5	Male	25	University	None	N12	Female	46	University	None
N6	Male	21	High school	None	N13	Male	52	High school	None
N7	Female	29	University	None	N14	Male	61	High school	None

EHR: electronic health records.

**Table 2.** Description of physicians participating in the expert interviews.

ID	Gender	Age	Medical specialty	Setting
Doc1	Male	46	General medicine and orthopaedics	Outpatient
Doc2	Male	37	General medicine	Outpatient
Doc3	Female	54	General medicine	Inpatient
Doc4	Female	36	Paediatrics	Inpatient
Doc5	Female	36	General medicine	Outpatient
Doc6	Male	56	Anaesthesiology and ELGA representative	Inpatient
Doc7	Male	54	Psychiatry	Inpatient and outpatient
Doc8	Female	30	Neurosurgical	Inpatient

## Step 0: Facilitators and barriers related to physicians as EHR gatekeepers

Eight factors related to the role played by physicians with regard to EHR usage were identified. The physicians seemed to have a gatekeeping role and they determined whether patients entered the process stages of EHR usage. If physicians expressed a negative attitude towards EHR, patients might not even enter Step 1 of EHR usage.

### Patient perspective

#### Networking with and between physicians

High perceived value for networking with and between physicians in a treatment process emerged as a facilitating factor. However, the lack of interoperability of systems presented as a substantial hurdle: ‘The inconsistency I see is that hospitals work with several systems, which means there can never be a baseline’ (U11, male, 53).

### Physician perspective

#### Cost–benefit analysis of EHR

At the meso-level, from the physicians’ point of view, there was currently no balanced relationship between the costs and benefits of the EHR system:

The benefits of products like ELGA can be immense (i.e., creating medical reports using text modules or automatic speech recognition), but you have to be able to fully exploit them . . . I have to honestly admit that our medical practice exploits 20–25% of what the product can do. (Doc1, male, 46)

#### Differences compared to the time before EHR systems

Finally, from the physicians’ perspective, there had been no noticeable differences since the introduction of the EHR system. Minimal visible changes were nevertheless noticeable: ‘Quite banal, there is simply less paper’ (Doc4, female, 36).

### Patient and physician perspective: Consistent views in relation to EHR

#### Previous contact points

Patients noted that previous contact points for EHR were predominantly negative, since general physicians communicated negatively about EHR. They also remembered flyers in the waiting rooms: ‘I can only remember one visit to my physician, and there were posters et cetera “Get out of ELGA”)’ (N1, male, 35).

Physicians expressed that previous contact points with patients in relation to EHR were relatively neutral, since most patients seemed to be unaware of the rollout plan and its steps or the ‘opt-out’ procedure of the EHR system in Austria: ‘First of all, most patients don’t even know that they are registered in the system and have to actively opt-out. For my practice, I can speak of 90–95% who have no idea about the conditions of participation’ (Doc1, male, 46).

#### Physicians as EHR gatekeeper

The physicians’ role as gatekeepers of EHR usage was classified as that of a facilitator who motivated and influenced patients: ‘I see the general practitioners as the best option to convince patients’ (U1, female, 29), who could make patients more aware of EHR. In addition, the general physicians act as a low-threshold information base for patients: ‘Exactly, it’s the physicians themselves. The patients trust them and if it works at the grassroots level, it gets transported upwards. This is an opportunity that should be used!’ (U6, female, 29).

The physicians’ view regarding the gatekeeper role here is somewhat different and calls for a clear boundary between medical and technical educational work: ‘Yes, I am a person of trust and also a gatekeeper *but* in medical terms in a treatment process’ (Doc1, male, 46). The experts saw the technical introduction and explanation for the use of the system as being the responsibility of a higher authority.

#### Attitude towards EHR of patients and physicians

From the patients’ perspective, a negative attitude and behaviour of physicians towards EHR was reasonable and attributed to perceived customer competition between physicians: ‘Personally, I could also think of it as “job security” because my physician has the exact information on my health data and therefore I am “bound” to him’ (N9, male, 54), and an increased effort for financing of and training on systems.

Attitudes towards the EHR system varied for both younger and older colleagues. Digital affinity seemed to foster a positive attitude towards EHR usage:

My partner in the group practice is 64 years old and both motivated and digitally literate. He believes that the medical practice needs a qualitative system (ELGA), but only because he has familiarised himself with it (he does his own research and online training) and also because he is convinced of its benefits. (Doc1, male, 46)

#### Privacy and security concerns of EHR

Bearing in mind the sensitivity of the exchanged data, patients found that privacy and security throughout the entire EHR usage process had to be comprehensively guaranteed: ‘You can’t afford to make mistakes at such a level where you work with sensitive data, so I trust the system’ (N11, female, 50).

However, experts in the physicians’ segment viewed patients’ privacy and security concerns rather critically, as, in their view, data exchange and networking happen without problems in many other realms of everyday life:

We should consider the things we do on a daily basis (points to the AppleWatch, social media). Payment via mBanking, shopping via AmazonPrime, sports via calorie tracking, et cetera, but as soon as it comes to ELGA, they don’t want that. (Doc5, female, 36)

Furthermore, experts did not report increased competition among colleagues through the existence of EHR systems, as competition exists with and without the EHR system: ‘No, because it has nothing to do with ELGA, whether you read and theoretically criticise the diagnosis in digital or in physical form’ (Doc1, male, 46).

#### Data sharing of EHR

On the macro-level, one issue emerged: data sharing. Data can be accessed regardless of time and place in critical medical situations, which was seen as positive. Moreover, there was uncertainty about which physician actually accessed one’s data, when and how: ‘Does everyone see everything about me, or do I actually know who is allowed to read what?’ (U12, male, 61).

From the experts’ point of view, the procedures and responsibilities for data sharing in an EHR system are not clearly defined: ‘There is also no standardised procedure here as to what has to be recorded in ELGA? Is it enough to record a diagnosis? Or should the medication be entered? Who decides/regulates this? Currently, no one’ (Doc7, male, 54).

## Step 1: Facilitators and barriers of awareness of EHR

If the physician has successfully acted as a gatekeeper in Step 0, the patient might enter the first awareness stage of EHR. In this phase, in Studies 1 and 2, facilitators and barriers could be identified without exception at the personal level (micro-level) from both perspectives.

### Patient perspective

#### Personal view of EHR

The personal view of EHR was identified as the holistic collection of all personal health data in electronic form. Patients without usage experience mentioned that EHR portals are troublesome when it comes to handling the opportunity to opt out of the system. For example, ‘I find it interesting that we are all automatically enrolled in the EHR system, and you have to go through the registration process before you are allowed to opt out’ (N2, female, 29).

#### Challenging or motivating points of contact

Some patients also perceived the previous contact points with EHR as more challenging than motivating (i.e. requiring digital competencies, lacking assistance). Both users and non-users referred to this experience as a challenge: ‘Exactly, that is the main problem. Nobody is digitally aware, but there’s no help available anywhere’ (U2, male, 30).

### Patient and physician perspective: Consistent views in relation to EHR

#### Attitude towards EHR of patients and physicians

The attitude towards EHR as an influencing factor for experts with low usage experience occurred mainly in a negative context: ‘I don’t see the EHR system as an advantage for the patient anyway, but rather for the healthcare system’ (Doc8, female, 30).

#### Information and EHR strategy

Finally, the most important inhibitor of awareness to use EHR on the personal level was a lack of information strategies for both target groups. The information available on EHR was perceived as insufficient, as was mentioned by users and non-users in the patient segment as well as by physicians: ‘The biggest area is undoubtedly the information gap that exists. Patients do not know what an EHR system is, what advantages it brings or what to do with it’ (N3, female, 23). Provision of information was viewed as insufficient from both the patients’ and the physicians’ perspectives.

## Step 2: Facilitators and barriers of willingness to use EHR

### Patient and physician perspective: Consistent views in relation to EHR

#### Information and EHR strategy

In Step 2 (willingness to use EHR), the views of patients and physicians were similar, as both felt poorly informed about EHR in general. One critical and omnipresent theme was the fear that EHR usage would be mandatory in the future for both patients and physicians. However, physicians did not currently see themselves as being able to utilise an EHR system fully: ‘How can I explain the EHR to a patient if I have no idea about the concept myself?’ (Doc3, female, 54).

## Step 3: Facilitators and barriers of usage experience with EHR

In summary, three facilitators and inhibitors were identified in the area of user experience on the micro-level. The same three factors were mentioned on the meso-level of the EHR system: usability, technical necessity and additional features.

### Patient perspective

#### Usability of EHR

The usability of collecting, saving and administrating EHR was perceived as a considerable barrier and challenging, as mentioned by users as well as non-users, who received initial training on the login process and use of the EHR system at the beginning of the focus group discussions: ‘The login-procedure, with path and various links and shortcuts, is much too complicated’ (N7, female, 29).

### Physician perspective

#### (Non-)Usefulness of EHR

There was a broad range of opinion with regard to the usefulness of EHR. Some physicians were not convinced of its usefulness: ‘Even in an emergency, primary care must function without an EHR system’ (Doc8, female, 30), whereas in contrast, others found EHR helpful in some respects: ‘For me, the medical aspect is in the foreground, avoiding unnecessary multiple treatments, such as medication or procedures (X-rays)’ (Doc4, female, 36).

### Patient and physician perspective: Consistent views in relation to EHR

#### EHR literacy

A new theme, termed ‘EHR literacy’ by the authors, emerged inductively. It refers to the specific competency necessary to be able to manage and use EHR effectively. High perceived EHR literacy could be classified as a facilitator of EHR usage. On the other hand, particularly in relation to the population of older people, low EHR literacy can impede effective EHR usage and negatively influence the treatment process with health providers: ‘It takes an immense amount of effort when a patient who is not digitally literate comes to see me at the clinic’ (Doc1, male, 46). Without the support of others (e.g. family members, friends, health professional (colleagues)), patients might be limited in their options to use the EHR system.

#### Information and EHR strategy

Building on the previously mentioned obstacles with regard to limited knowledge of EHR, one of the factors dealt with possible mediated information strategies on a personal level: ‘Nowadays, it makes sense to use all the channels you have to achieve a broader distribution’ (N11, female, 50).

Physicians have provided some current examples to achieve improved information strategies for EHR systems. For instance, the open discussions on television during the first peak of the COVID-19 pandemic in 2020 in Austria were described as valuable: ‘access requirements and privacy terms from the EHR system’ (Doc5, female, 36).

#### Technical necessity for use of EHR

Similarly, the technical necessity for secure access and a transparent EHR usage process was perceived as indispensable: ‘Especially for EHR systems, which handle sensitive data, a “strict” entry must be mandatory’ (U12, male, 61).

#### Additional features of EHR systems

Another reoccurring theme was thinking about future possible additional features of the EHR system that would lead to a higher usage rate. For example: ‘An example would be the constant communication of current information on diseases, with symptoms, and treatments, et cetera’ (N1, male, 35).

## Discussion

The contribution of the present study is three-fold. First, from a public health perspective, it contributes to a better understanding of the differing perspectives of patients and physicians with regard to facilitators and barriers of EHR usage. Second, distinct facilitators and barriers could be identified in different stages of the entire EHR usage process (from awareness raising, to willingness to use and the existing user experience, underlined by the physicians’ role as EHR gatekeeper). Third, the themes emerged on three different levels: the micro-level (individual level), the meso-level (level of the EHR system) and the macro-level (level of the health system). Most themes discussed focused on the micro-level. A lack of awareness, as well as a lack of knowledge about EHR, was seen as the main barrier. Greenhalgh (2008) explained that patients’ awareness impacts on the way they perceive and process information as well as on their behaviours. In addition, most patients in this study were unaware of the opt-out scheme in the Austrian EHR system, which allows patients to restrict their health information or fully opt out of the programme (ELGA GmbH, 2021). This discrepancy highlights the complexity of the EHR system and indicates the existence of barriers that hinder patients from actively using EHR. Interestingly, this fact was not detected in the previous literature.

EHR literacy was identified as a necessary prerequisite to handle the complex, though classified as secure, login procedure and to be able to navigate its features. Torrens and Walker (2017) emphasised the importance of empowering patients of all ages to manage their EHR, ensuring that special population groups, such as older people, are consistently represented. These findings broadly support findings in previous studies, namely that digital competencies and applying the knowledge gained can help patients better understand medical conditions and treatment plans, make informed decisions and take proper action (Chen and Decary, 2019). To realise these advantages, widespread acceptance and use of EHR by healthcare actors are required (Hanna et al., 2017).

The identified facilitators and barriers were assigned to different phases of the EHR usage process. Strongly reported in the first phase of (1) awareness were facilitators, such as sufficient and continuous information and training for all health actors in a treatment process (i.e. patient, physicians). In line with previous findings about the importance of ensuring the handling with EHR, improved patients’ and physicians’ (Henry, 2006) awareness of EHR is critical before an applied system is recommended (Mossaed et al., 2015). With the help of measures through various communication channels, EHR can be made more appropriate (Riordan et al., 2015). Furthermore, self-initiative and step-by-step self-learning from the system are the basis for mastering EHR. However, negative experiences with contact points during the first step impeded further willingness to use EHR (Goel et al., 2011).

The lack of communication strategies targeted to health actors (i.e. younger and older patients, patients and physicians) seems to prevent people from engaging in EHR usage. The information situation of EHR relates not only to the phase of (2) willingness to use, but also to (3) the usage experience prior to information strategies. This finding is consistent with other studies, which emphasise that patients feel uninformed about EHR (Riordan et al., 2015). A future factor ‘additional features’ (i.e. check-up reminder, news of current health information, side effects of medication) in the EHR system, to maximise patients’ and physicians’ intention to use, could be identified to streamline management of health information in this study. Murphy et al. (2019) and Guo et al. (2017) identified suboptimal design features and workflows as reasons for not using EHR.

General physicians or other physicians were specified as a kind of gatekeepers for EHR usage from the patients’ perspective. It is well known that general practitioners act as ‘gatekeepers’ in their role of offering patients access to specialised care in direct-access systems (Whitten and Kuwahara, 2004; Yellowlees et al., 2018). The gatekeeper is defined as the ‘individual of first contact, who controls the flow of information to others within the company’ (Lau et al., 2003, cited by Bierma and Waterstraat, 2008: 697). Sripa et al. (2019) could demonstrate that gatekeeping was on the one hand related to better quality of care, but on the other hand it resulted in fewer hospitalisations. We use the term ‘gatekeeper’ in relation to the information delivered on a new technology similar to the approach taken by Lauckner and Whitten (2016). A broad literature base exists in marketing referring to innovation adoption (i.e. Bierma and Waterstraat, 2008; Smith et al., 2020).

Physicians participating in our Study 2 do not feel responsible for the dissemination of the EHR system and assigned the responsibility for the technical introduction of the system to some kind of higher authority (i.e. the public sector) since basic knowledge is lacking among physicians. Furthermore, from the patients’ perspective, physicians are seen as responsible for creating comprehensive awareness (White and Danis, 2013). This facilitating factor regarding EHR usage was not identified clearly earlier. Past studies have focused primarily on the medical aspects for the duration of the treatment period rather than on ‘IT technical’ support for patients.

Nevertheless, the study findings report that the current physicians’ attitude towards EHR is negative and inhibits further actions in the usage process (Lakbala and Dindarloo, 2014). Furthermore, a certain level of basic knowledge must be present on the part of the physician to fully exploit the possibilities in dealing with EHR (Voran, 2017).

To the best of our knowledge, this is the first study that takes a holistic approach to receive in-depth knowledge on barriers and facilitators of patients’ and physicians’ EHR usage along the entire usage process.

### Limitations and implications for future research

This research had some limitations. Focus group participants were distributed disproportionally with regard to gender and educational background, which may have reduced the generalisability of results. Nonetheless, the research has highlighted promising areas for further study, with this study providing the first step towards a deeper understanding of facilitators and barriers along the entire EHR usage continuum. However, our results should be confirmed with a larger and more representative sample reflecting the diversity of the Austrian healthcare system. In countries with similar healthcare systems, our research could serve as an anchor for a similar holistic view. It could also be interesting to analyse our coding system with subsections of the EHR system, such as the Digital COVID Certificate, created by the government.

## Conclusion

This study has provided a holistic understanding of patients’ and physicians’ views of the facilitators and barriers during the entire EHR usage process. In particular, we have highlighted the critical function of physicians as gatekeepers who played a key role in the use of EHR by their patient base. Results showed that patients’ awareness and knowledge of the Austrian EHR system mitigated their EHR usage due to a lack of effective communication at the interpersonal, interactive and mass media levels (i.e. login procedure, opt-out scheme). Furthermore, we confirmed that for physicians, perceived risks outweighed the benefits of EHR use. Qualitative evidence from patients and physicians has underlined the importance of involving all the health actors in the entire EHR usage process. Awareness raising leads to willingness to use, which is further facilitated by actual usage and the beneficial consequences of EHR usage. Our study offers new insights that lead to a more critical elaboration of mediated health communication, including improved patient and physician usage of EHR.
